# Historical, Observed, and Modeled Wildfire Severity in Montane Forests of the Colorado Front Range

**DOI:** 10.1371/journal.pone.0106971

**Published:** 2014-09-24

**Authors:** Rosemary L. Sherriff, Rutherford V. Platt, Thomas T. Veblen, Tania L. Schoennagel, Meredith H. Gartner

**Affiliations:** 1 Department of Geography, Humboldt State University, Arcata, California, United States of America; 2 Department of Environmental Studies, Gettysburg College, Gettysburg, Pennsylvania, United States of America; 3 Department of Geography, University of Colorado, Boulder, Colorado, United States of America; DOE Pacific Northwest National Laboratory, United States of America

## Abstract

Large recent fires in the western U.S. have contributed to a perception that fire exclusion has caused an unprecedented occurrence of uncharacteristically severe fires, particularly in lower elevation dry pine forests. In the absence of long-term fire severity records, it is unknown how short-term trends compare to fire severity prior to 20^th^ century fire exclusion. This study compares historical (i.e. pre-1920) fire severity with observed modern fire severity and modeled potential fire behavior across 564,413 ha of montane forests of the Colorado Front Range. We used forest structure and tree-ring fire history to characterize fire severity at 232 sites and then modeled historical fire-severity across the entire study area using biophysical variables. Eighteen (7.8%) sites were characterized by low-severity fires and 214 (92.2%) by mixed-severity fires (i.e. including moderate- or high-severity fires). Difference in area of historical versus observed low-severity fire within nine recent (post-1999) large fire perimeters was greatest in lower montane forests. Only 16% of the study area recorded a shift from historical low severity to a higher potential for crown fire today. An historical fire regime of more frequent and low-severity fires at low elevations (<2260 m) supports a convergence of management goals of ecological restoration and fire hazard mitigation in those habitats. In contrast, at higher elevations mixed-severity fires were predominant historically and continue to be so today. Thinning treatments at higher elevations of the montane zone will not return the fire regime to an historic low-severity regime, and are of questionable effectiveness in preventing severe wildfires. Based on present-day fuels, predicted fire behavior under extreme fire weather continues to indicate a mixed-severity fire regime throughout most of the montane forest zone. Recent large wildfires in the Front Range are not fundamentally different from similar events that occurred historically under extreme weather conditions.

## Introduction

The social, environmental and fiscal costs of wildfire have escalated dramatically over the last few decades [1]–[2]. The costs associated with recent wildfires are particularly high in the arid mountain West, where residential structures abut or intermingle with wildland vegetation (Wildland-Urban Interface - WUI) and the exurban population has grown rapidly in recent decades [3]. Large fire events in the 1990s and early 2000s in the western U.S., particularly in lower elevation, relatively dry-pine forests, have contributed to widespread concern that fire exclusion has caused an unprecedented threat of uncharacteristically severe fires in these ecosystems [4]–[5]. Broad-scale monitoring of fire severity from satellite imagery since ca. 1984 shows a significant trend towards increased severity only in parts of the Southwest [6], yet findings are varied in other studies depending on the spatial scale, selected data types, and the location of study (e.g. Pacific West) [7]–[12]. In the absence of longer broad-scale records of fire severity, it is unknown how such short-term trends compare to fire severity prior to fire exclusion. In the context of debate about the potential effects of fire exclusion on modern fire regimes and their departures from historical fire regimes (i.e. prior to fire exclusion) [4], [13]–[15], there is a critical need for research on whether the severity and other characteristics of modern fires depart from historical fire regimes for specific ecosystem types at broad landscape scales. The current study compares reconstructed historical fire severity, observed fire severity in recent fires and modeled fire severity from current fuel structures, and discusses the consequences of fire regime changes for management options under expected future climate in the Colorado Front Range.

Development of management goals and adaptation options in fire-prone ecosystems interfacing with the WUI is inextricably related to quantifying the range of variation of a set of ecological patterns and processes exhibited naturally or under human influences during a specified historical period [16]–[17]. This widely used approach, called the historical range of variability (HRV) framework uses historical ecological data to test hypotheses about the drivers and mechanisms of contemporary and future ecological change [18]. Management using the HRV framework assumes that ecosystem resilience (the ability to recover quickly) is reflected in observed ranges of past vegetation and fire dynamics. Retrospective ecological studies are considered essential for understanding likely consequences of climate change on future fire and landscape dynamics [18]–[19]. While there has been a shift away from using reference conditions as default for fire and forest management goals in relation to future climate change [19]–[20], HRV still provides the most viable framework for understanding the sensitivity of ecosystem resilience and ecological integrity to changes in fire regimes [21]. Ecological integrity has been defined as a measure of the composition, structure, and function of an ecosystem in relation to the system's natural or historical range of variation, as well as changes caused by humans [22]–[23]. A key research objective in the context of fire-prone landscapes in the U.S. West is the assessment of the historical role of fires of varying severities on the resilience and integrity of current ecosystems and their potential consequences for ecosystem services valued by humans [21].

Fuel reduction is currently the dominant management tool for reducing the likelihood of high-severity fire in the contexts of ecological restoration and mitigation of climate change impacts [24]–[26]. This dual approach (forest restoration and fire mitigation) assumes that the probability of severe fire occurrence has increased to uncharacteristic levels during decades of fire suppression in western forests [4], [27]–[34]. Guided by this assumption, over 190 million acres of public land have been identified as “unnaturally dense” with an increased likelihood of catastrophic wildfires [24], [26]. In response to such concerns, both forest managers and communities have begun to develop strategies to alleviate the potential impacts of wildfire [35], and millions of hectares of forest lands have been treated in recent decades [36]. The goals of fuels reduction to decrease the likelihood of severe wildfires and restore historical forest structure and species composition are complementary in ecosystems where fuels and fire severity have increased, yet are incompatible elsewhere and threaten ecosystem integrity and ecosystem services [37]–[43].

In the Colorado Front Range, tree-ring evidence, historical landscape photographs, and General Land Office surveys demonstrate that the historical (i.e. pre-1920) fire regime of ponderosa pine and mixed-conifer forests included low-severity fires (i.e. non-lethal to large fire-resistant trees) as well as high-severity fires (i.e. killing >70% of canopy trees) [13], [44]–[54]. There is a broad consensus that most of the montane zone of Colorado was characterized by fire regimes of mixed severity, including some component of high-severity fires [55]. However, a better understanding of which habitats across this heterogeneous landscape were explicitly affected predominantly by low-severity or higher-severity fires, or a combination of both, is necessary for determining where woody fuels have become uncharacteristically abundant as a consequence of fire exclusion [37], [56].

**Figure 1:**
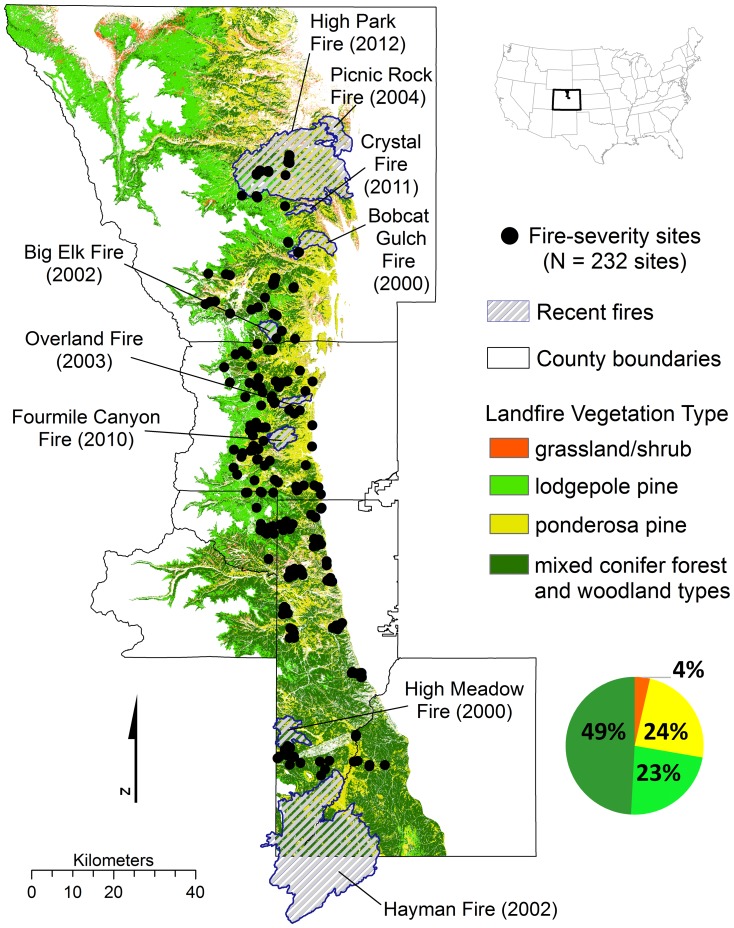
Study area in north-central Colorado, USA. The map includes the montane zone by cover type (LANDFIRE Existing Vegetation Type), 232 sites sampled for historical fire severity, and recent wildfires used for comparison and verification of fire behavior modeling. County boundaries (solid black lines) from north to south are of Gilpin and Clear Creek counties and the mountainous western regions of Larimer, Boulder, and Douglas counties, and Jefferson County.

Thus, in this research we examine changes in fire regimes across 564,413 ha of the montane forest zone of the Colorado Front Range (Figure 1). We compile and analyze new and existing datasets to refine the spatial resolution and expand the geographic scope of retrodicted (reconstructed) historical fire regimes in comparison with present wildfire potential. We address two main questions about the montane forests of the Colorado Front Range: 1) What areas and landscape characteristics were characterized by an historical fire regime of predominantly low-severity or mixed-severity (which includes moderate- or high-severity fires)? 2) How does historical fire severity compare with observed severity of large wildfires (since 2000) and modeled potential wildfire behavior across the landscape? Finally, we discuss how these comparisons can inform fire mitigation and ecological restoration under expected climate change.

## Methods

### Study area

The study area is located in the north-central Front Range of Colorado, bounded by six counties and the extent of the montane zone between 1800–3000 m to the east and west (Figure 1). Within the lower to upper montane zones, the mean annual precipitation ranges are ∼35.6 to 51 cm and the mean annual temperature ranges are 11°C to 2.4°C, respectively [57]. The lower montane zone (∼1800 to 2200 m) comprises primarily pure ponderosa pine (*Pinus ponderosa*) on south-facing slopes and a mixture of ponderosa pine and Douglas-fir (*Pseudotsuga menziesii*) on north-facing slopes. The upper montane zone (∼2200 to 3000 m) is composed of ponderosa pine stands on south-facing slopes and more dense stands of ponderosa pine and Douglas-fir on north-facing slopes along with lodgepole pine (*Pinus contorta*), aspen (*Populus tremuloides*) and dispersed limber pine (*Pinus flexilis*) trees at higher elevations. The LANDFIRE existing vegetation type (EVT) layer was used to delineate the montane study area (564,413 ha) within 1800–3000 m because it represents the most up-to-date and detailed cover type classification available across land ownerships in the region, which is proportionally represented by the following EVT types: ponderosa pine (24.2%); lodgepole pine (23.1%); mixed montane conifer forest and woodland types (49.2%); and intermixed with pixels classified as lower montane-foothill grassland (<0.1%) or shrubland (3.5%) (Figure 1; EVT types included in the study are listed in Table S1). However, the accuracy of the LANDFIRE EVT classification at field sites was relatively low (see RESULTS).

During the period from 1980 to 2011, 48 fires burned over 100,000 ha along the Front Range causing severe damage to property and infrastructure in the WUI [2], [58]–[59]. These large fires can occur any time of year and typically burn when wind speeds are high and weather conditions are dry, which is common along the Front Range. During these types of conditions, fire suppression is typically ineffective and fires often escape initial suppression efforts [2].

### Field sampling

We used a combination of existing datasets (141 sites) [52]–[53], [60]–[62] and newly sampled datasets (91 sites) for a total of 232 sites with information on forest structure (tree age structure) and fire history (tree-ring fire-scar records) in montane forest types. The majority of sites were sampled in a stratified-random design and all sites were selected in relative proportion to their cover type within the study area, allowing inferences from our site-level datasets to reflect trends across the 564,413 ha montane study area. Because sites were dispersed over the broad regional area of montane forests we do not extrapolate the spatial spread of fire severity between sites, or determine if fires in the same year at multiple sites were from a single ignition or multiple ignition sources. We rejected sites with evidence of past logging or signs of other major anthropogenic disturbance (i.e. mining). Our sampling goal, similar to previous site-level studies of historic fire regimes in the region [51], [53], was to identify the predominant structural influence of historical fires at each site (fire-severity regime as explained below). Understanding the historical fire regime and the effects on forest structure without major Euro-American influences provides the natural range of conditions. The interactive effects of logging and/or other anthropogenic disturbances are important, but beyond the scope of our study. Laboratory (tree-ring) analysis of new sites followed the same procedures used in prior studies for analyzing fire-scar samples, stand age and size structure [51]–[53], [60]–[62]. The criteria used to characterize the historical fire severity at each site were derived from the results of these prior studies and are described below (*Site-level classification of historical fire severity*).

#### Stand-level sites

At 120 of the 232 sites [51]–[53], [60]–[62] (and unpublished new data), we sampled in areas of relatively consistent (unvarying) forest structure and physical environment with the extent of sampling area varying in size from 10 to 232 ha according to tree density and the extent of the homogeneous stand structure. At 44 of the stand-level sites, fire-scarred trees were systematically sampled [60], [61], fire-scarred trees and fire scars on each fire-scarred tree were tallied, and forest structure was evaluated based on the same protocol used by Sherriff and Veblen [52] (see Tables 1–2). At the remaining 76 stand-level sites, the sampling goal was more intensive. In addition to systematically sampling fire-scarred trees throughout the site, at least 50 of the closest trees (≥4 cm dbh) at a constant distance along randomly located transects were cored, and at least five of the largest and oldest characteristic live or dead trees were selectively cored to ensure large (and the oldest characteristic) trees were represented in our sample. In addition, forest structure (size, decay class, standing/down, distance from transect point), seedling (<30 cm height) and sapling (>30 cm height and <4 cm diameter) data were tallied by species along belt transects. To estimate the ages of trees too small to core (<4 cm in diameter) and to estimate the number of rings missed due to coring height (approximately 20 cm above the root-shoot boundary), 341 juvenile trees, reflecting the range of tree species within the sampling sites (in order of abundance - ponderosa pine, Douglas-fir, lodgepole pine, aspen and limber pine), were cut at sites of different elevation and aspect. Relatively open sites were selected to mimic post-fire growth conditions. Permits for sampling were issued by Rocky Mountain National Park, USDA Forest Service, and county and city open space land management agencies. Sampling did not involve protected or endangered species. For sites where seedlings were not collected, the median age-to-coring height was used from a site of similar elevation and aspect (across all sampled sites: median of 4 years – ponderosa pine; 9 years – Douglas-fir; 8 years – lodgepole pine; 1 year – aspen; 9 years – limber pine). To correct for years missed due to missing the pith, we followed Duncan's [63] procedure. Cores with more than 20 years estimated to pith were excluded.

**Table 1:** Definitions of historical fire severity terms.

Term	Definition of fire effects
**Historical high-severity fire**	A fire that had high mortality of live, standing vegetation (<20% of the sampled trees survived the fire) and high tree establishment (>80% of the sampled trees) following the fire.
**Historical low-severity fire**	A fire that had low to no mortality of live, standing vegetation (>80% of the sampled trees survived) and low to no establishment (<20% of the sampled trees established following the fire).
**Historical low-severity fire regime**	Dominated by frequent (Mean Fire Interval <30 years), non-stand replacing fires within a stand (∼100 ha) that leave multiple fire scars on individual trees throughout the stand and kill young seedlings and subcanopy trees while maintaining open, low-density stands of fire-resistant canopy trees.
**Historical moderate-severity fire**	A fire that had effects that were intermediate between low and high severity.
**Historical mixed-severity fire regime**	Varied fire effects that included low-severity, non-stand replacing fire to high-severity, stand- (or canopy) replacing fire both within stands and across landscapes, often in relation to topography.

These terms may have different meanings in the literature depending on the context in which they are used.

**Table 2:** Large fires in the montane study area from 2000–2012.

Fire	Year	Size	Fire Severity (%)*			
		(ha)*	Unburned/Low	Low	Moderate	High
Bobcat Fire	2000	3688	30.5	16.8	22.6	30.1
High Meadows Fire	2000	3884	21.7	37.8	32.0	8.5
Big Elk Fire	2002	1741	42.1	20.0	17.1	20.8
Hayman Fire	2002	52,016	21.1	12.7	22.5	43.7
Overland Fire	2003	1244	27.9	12.9	22.6	36.6
Picnic Rock Fire	2004	3626	27.6	37.7	22.6	12.1
Four Mile Fire	2010	2285	11.0	25.6	34.3	29.1
Crystal Fire	2011	914	22.9	38.5	26.9	11.7
High Park Fire	2012	34,905	20.8	29.7	26.9	22.5

* Total area burned and fire severity percentages within each fire perimeter (MTBS values 1–4; 1 =  unburned/low, 2 =  low, 3 =  moderate, and 4 =  high). See Figure 1 for large fire perimeters used to compare historical and observed fire severities within the study area.

#### Plot-level sites

The remaining 112 sites with information on forest structure and fire were smaller in size (3-ha plots) and randomly located from a 1-km grid throughout the CFR study area [52] (and new unpublished data). Sites with abundant logging or other human disturbances were rejected and an adjacent location was sampled. At each random plot-level site the number of fire-scarred trees and fire scars on each fire-scarred tree were tallied, fire-scarred trees were cored to estimate fire dates [64], forest structure was documented using an existing protocol [52] (see Tables 1–2), and approximately 10 cores were taken for reconstructing tree ages.

### Analytical procedures

#### Site-level classification of historical fire severity

For this analysis, we use common and broad definitions of fire-severity regimes for montane forests of the Colorado Front Range that are relevant to forest structure and management (Table 1). Low-severity fire regimes are dominated by frequent (Mean Fire Interval – MFI <30 years), non-stand replacing fires within a stand (∼100 ha) that leave multiple fire scars on individual trees throughout the stand and kill young seedlings and subcanopy trees while maintaining open, low-density stands of fire-resistant canopy trees. Mixed-severity regimes have varied fire effects that include low-severity, non-stand replacing fire to high-severity, stand- (or canopy) replacing fire both within stands and across landscapes, often in relation to topography [40], [47]–[48], [51]–[53], [65], [66]. High-severity fires occur less frequently (MFI >35–100+ years) than non-stand replacing fires, leave only small or no patches of pre-fire remnant trees, result in few to no fire scars on individual trees throughout the stand, and often initiate recruitment of a new cohort of canopy trees [51]–[53], [55].

First, we classified the severity of fires at each (stand- and plot-level) site based on their influence on forest structure. We focused on fire dates recorded by fire scars on at least two trees per site (spreading fires) in the same year prior to 1915 (ca. effective fire exclusion). Next we used two primary metrics, along with the dates and frequency of spreading fires, for characterizing the severity of fires at each site – the percentage of remnant trees (proportion of the trees older than a spreading fire date) and the percentage of tree establishment (proportion of the trees that established within 40 years after a spreading fire). These criteria follow the approach described in prior studies in the region [51], [53], correspond to classification of fire severity in other studies [65]–[66], and are summarized in Table 1. Specifically, individual fires were classified as low severity when ≥80% of the trees that were sampled had survived the fire (based on remnant tree ages) and ≤20% of the trees established following the fire. Fires were classified as moderate severity when 21–79% of the trees survived/established following the fire. Fires were classified as high severity when <20% of the trees that were sampled had survived the fire and >80% of the trees established following the fire. We excluded fires where no tree establishment followed within 40 years, assuming there was no structural impact or burning within the site [53]. Individual fires that occurred prior to subsequent mixed-severity fire, and were not associated with a pulse of establishment (>20% trees/site), were not included in the analysis of fire severity. We recognize that the estimate of fire effects for fires that occurred prior to a mixed-severity fire is less reliable because of subsequent mortality of older trees with time and/or subsequent disturbance. Post-fire tree establishment is not a direct measure of fire severity, and can vary significantly among species and in relation to seed availability and climate conditions, but it provides corroborative evidence of fire severity in combination with remnant trees, which provide a more direct measure of fire severity. Both metrics, pre-fire remnant trees and post-fire tree establishment, have uncertainties for evaluating fire severity and are not a perfect complement, but used in conjunction with fire dates and integrated into measureable metrics represent a robust way to reconstruct historical fire severity in montane forests (see [53] for more on methodological limitations and recommendations).

Based on the fire severity classifications, we assigned a predominant fire regime at each (stand- and plot-level) site based on the following criteria of cumulative fire effects over time. Sites characterized by frequent, non-stand replacing fires (MFI <30 years) that left multiple fire scars on individual trees throughout the site and without evidence of higher-severity fire were classified as having only low-severity fire regimes. Low-severity sites identified without historical evidence of moderate- or high-severity fire are assumed to have had only low-intensity (surface) fire and low to no canopy mortality. Sites characterized by varied fire effects that included canopy-replacing fire within sites were classified as having mixed-severity regimes (see [51]–[53] for within site sampling protocols). Mixed-severity fire regimes include low-, moderate- and high-severity fires where sites may illustrate different severities over time or space depending on weather, climate and stand conditions. Individual sites were differentiated based on evidence of low- and moderate-severity fire effects (no evidence of high-severity fire with <20% the stand age preceding a single fire) or low- to high-severity fire effects. Sites identified with either (low- and moderate-severity fire effects or low- to high-severity fire effects) were classified as a mixed-severity fire regime. Mixed-severity sites identified with moderate- or high-severity fire are assumed to have had components of intense fire behavior and canopy mortality, but varied in the extent of canopy mortality.

#### Landscape-level modeling of historical fire severity

Based on the fire severity classifications at the 232 field sites, we modeled historical fire-severity regime across the study area using potential predictor variables derived from two sources: 1) terrain variables from a 30-m digital elevation model (DEM); and 2) LANDFIRE biophysical variables. Environmental conditions at each of the 232 sites were classified in terms of the median elevation, slope steepness, aspect, cover type, distance to grassland, distance to ravine and the associated fire regime type. Elevation, slope steepness, aspect (arcsine and sine transformations) and slope curvature were all derived from the 30-m DEM. Slope curvature (concavity to convexity) and hillslope position are related to soil depth, texture and potential soil moisture [67]. Ravine drainages were delineated from the 30-m DEM using Arc GRID (ESRI 2002) hydrologic terrain modeling [68] and distance to ravine drainage was calculated. Distance to grassland was calculated as the distance to the edge of grassland areas of at least 0.1 ha.

Classification and regression trees (CART; *n* = 5000 trees) were used to identify explanatory variables with the most power to predict fire severity type (RandomForests Software, Salford Systems). CART has been increasingly used in ecological studies that require nonparametric techniques to explore complex, hierarchical interactions among variables [69]–[71]. We evaluated predictor variables for three response variables (historical low-, moderate- and high-severity fire) and a binary response variable (historical low- or mixed-severity fire) using all 232 sites to identify complex and non-linear interactions between variables that could be mapped across the study area (e.g. [71]). A 10-fold cross-validation process was used to avoid over fitting. The regression tree size with the smallest error was recorded for each run. The average tree size was used to prune the original tree (from the complete dataset), which tends to minimize complexity and results in a more generalizable tree [72]. There was strong overlap in the range of environmental conditions at sites with evidence of historical moderate and high fire severities. For this reason, we used the results evaluating the binary response variable (historical low- or mixed-severity fire) across all 232 sites for this study to produce a predictive map of the historical fire severity across the CFR study area in a GIS (ESRI ArcGIS 10.1). We identified the optimal model as the one with the fewest variables that best predicted the occurrence of sites with historical low and mixed severities. Model performance was evaluated using the overall percentage correctly classified (PCC) and area under the receiver operating characteristic curve (AUC) statistic. An AUC value of 0.5 indicates the prediction accuracy of sites picked at random, whereas 1.0 indicates perfect classification accuracy. AUC values of 0.7, 0.8, and 0.9 indicate fair, good, or excellent accuracy, respectively.

#### Comparison of historical fire severity to observed fire severity

Observed fire severities from nine large fires that occurred since 2000 (Table 2; Figure 1), including a range of fire severity, were compared with the spatially-explicit map of historical fire severity (Table 3). The ‘observed’ severity data came from the Monitoring Trends in Burn Severity (MTBS) program, which provides maps of the perimeters and severity of all fires larger than 1000 ha in the western United States from 1984-present [73]. MTBS thematic burn severity classification maps for each fire were used to evaluate the observed fire severity (unburned/low, low, moderate, and severe) and compared with the spatially-explicit map of historical fire severity within the study area (ESRI ArcGIS 10.1). We recognize these two datasets (observed fire severity and historical fire severity) are derived from different sources of information, each with their own limitations. Thus, our analysis focused on a summary comparison of the proportion of area burned by observed fire severity type compared to historical fire severity of the same area as a broad index of departure from historical fire regime reflecting landscape-level changes rather than a fine-scale (pixel-by-pixel) comparison. For each of the nine fires, we tested for differences (in the number of pixels) between the observed amount of low-severity fire (MTBS unburned/low and low severity classes) compared to the expected (historical low-severity) using χ^2^ tests (IBM SPSS 20).

**Table 3:** Comparisons of historical fire severity, observed fire severity, and modeled potential fire behavior.

Historical	Observed (MTBS thematic burn severity classifications)	Modeled (FlamMap output)
Low-severity fire only	Unburned to low severity, Low severity	Surface fire, e.g. median fireline intensity <33,000 kW/m
Mixed-severity fire: evidence of varied severities from low-, moderate- to high-severity fire	Moderate severity, High severity	Crown, torch, e.g., median fireline intensity >33,000 kW/m

#### Comparison of historical fire severity to modeled fire severity

The spatially-explicit model of historical fire severity was overlaid with a model of fire potential under extreme (99^th^ percentile) weather conditions to identify possible landscape-scale changes of historical low- and mixed-fire severity in the entire montane study area (564,413 ha; Table 3). We modeled potential fire behavior using FlamMap 3.0, a fire modeling system that predicts instantaneous fire behavior under fixed weather and fuel conditions (i.e. it is a static rather than dynamic model). FlamMap requires the following inputs: elevation, slope, aspect (derived from a 30 m USGS DEM); and percent canopy cover, stand height, canopy base height, canopy bulk density, and Scott and Burgan [74] fuel models (all from the LANDFIRE project [75]). Canopy cover and canopy base height values were adjusted downward on the recommendation of the LANDFIRE project [75] and a comparison of field-derived fuel layers in the study area [76]. The modeling procedure takes place in two steps (described in detail in [77]–[78] and summarized here). First, fuels are conditioned based on specified weather conditions. We used wind/weather conditions during the primary fire season (June–September) derived from four Remote Automated Weather Stations (RAWS) that span the study area and have the longest record: Redfeather (station 050505; 1964–2007), Estes Park (station 050507; 1964–2007), Corral Creek (station 051804; 1968–2007) and Bailey (station 052001; 1970–2007). In the second step, FlamMap calculates fireline intensity and crown fire activity using a suite of surface and crown fire models [79]–[82]. Fireline intensity is a measure of energy released per unit length along the flaming front of a fire (kW/m). Crown activity (a binary variable representing surface fire or active/passive crown fire) was calculated using the Scott and Reinhardt [83] method. To address how historical fire severity compares with modeled potential wildfire behavior, we overlaid the areas modeled as historical low-severity with potential wildfire behavior (fireline intensity and crown fire) assuming that areas of higher fireline intensity (e.g.>33,000 kW/m) and crown fire today indicate a shift from the historical fire regime to one of higher potential for crown fire and canopy mortality (>33,000 kW/m and >40,000 kW/m are the approximate median fireline intensity values for moderate and high severity fire, respectively, for all verification fires with variable severity described in the next section; Table S1). A crown fire may or may not be lethal to all dominant vegetation, and a crown fire may be continuous or may occur in patches within a mixed-severity burn. Mixed-severity fires, by definition, have components of intense fire behavior and canopy mortality, and high fireline intensity and active crown fire would be within the HRV. Our focus is on the areas of greatest change that may have shifted from low severity to potential high fire intensity or crown fire today. These fire behavior outputs were chosen because they are commonly used, interpretable, and can be generally compared to historical fire severity.

The wildfire modeling utilized the 99^th^ percentile monthly weather conditions from ca. 1964–2007 during the June-September fire season derived from four RAWS stations (listed above). Starting with a fixed southwest wind of 74 km/h, which is the 99.9th percentile daily wind speed during the primary fire season (June–September, ca. 1964–2007), we used the WindNinja program to refine wind speeds based on topography. FlamMap's input variables and modeling procedure follow Platt et al. [78] and were verified for a level of realism by comparing observed fire severity from seven major fires that occurred since 2002 (see the next section; Table 2).

To place historical 99^th^ percentile conditions into context, we first compared them to the conditions of past fires and then to predicted future climate. During the Hayman Fire (06/08/2002), temperature and relative humidity reached up to the 98^th^ and 99^th^ percentiles, respectively (Cheesman RAWS station). Similarly, during the High Park Fire (06/09/2012), both temperature and humidity were up to the 99^th^ percentile (Redstone RAWS station). The RAWS stations recorded wind gusts over 56 kph during the High Park Fire and over 80 kph during the Hayman Fire. We make the assumption that the 99^th^ percentiles are of comparable weather conditions between historical and contemporary time periods.

We then compared average monthly temperature in the study area during the June-September fire season to two climate model runs used in the IPCC Fourth Assessment Report [84]–[85]: (1) climate conditions in 1964–1999 from the Climate of the 20th Century (20C3M) model run and (2) climate conditions in 2064–2099 from the SRESA2 scenario. During the June–September fire season in 1964–1999 (20C3M model run), the 99^th^ percentile monthly temperatures were 24°C, and the 50^th^ percentile monthly temperatures were 19°C. During the June–September fire season from 2064–2099 (SRESA2 scenario), the 99^th^ percentile temperatures are predicted to increase to 30°C, and the 50^th^ percentile temperatures are predicted to increase to 26°C. The 99^th^ percentile temperatures from 1964–1999 are predicted to become 39^th^ percentile temperatures under the SRESA2 scenario. It is thus reasonable to say that the 99^th^ percentile conditions described in this study may become average conditions in 50 or more years from now.

#### Verification of modeled fire behavior

To assess the realism of the FlamMap models described above, we compared modeled fireline intensity and crown fire class to observed fire severity of the seven regional fires that occurred since 2002, the year for which the LANDFIRE (fuels) data is valid (Table 2). Within the perimeter of each fire, we generated randomly placed points, and at each point calculated median fireline intensity and percentage active and passive crown fire within four MTBS classes (unburned to low, low, moderate, and high severity; Table S1; ESRI ArcGIS 10.1). There are limitations to such a comparison. In an actual fire, intensity varies minute by minute depending on factors such as wind, relative humidity, fuel, and fire suppression. In contrast, FlamMap estimates intensity for an instant in time across an entire landscape for the generic “extreme conditions” described in the paper. Thus, while we cannot comprehensively validate a fire we can check whether, overall, areas identified as severely burned by MTBS correspond to higher modeled fireline intensity and crown fire. Kruskal-Wallis tests (IBM SPSS 20) were used to determine if the distribution of fireline intensity and percentage crown fire were significantly different between MTBS classes at the *p* = 0.01 level.

## Results

Across all 232 field sites, cover type was dominated by a single species (≥80% of the canopy trees >4 cm dbh) of ponderosa pine (42.2%–98 sites) and lodgepole pine (8.6%–20 sites), and co-dominated (<80% of the canopy trees a single species) by mixed-conifer types (49.2%–114 sites). Considering pure ponderosa pine together with mixed-conifer sites, 61.2% (142) of the 232 sites were dominated by ponderosa pine and the remaining sites were dominated by Douglas-fir (20.6% and 48 sites), lodgepole pine (15.5% and 36 sites), and aspen (2.6% and 6 sites). The overall accuracy of the LANDFIRE EVT classification was relatively low with 39.2% of the 232 sites misclassified: 31 pure ponderosa pine, 5 lodgepole pine and 55 co-dominated mixed-conifer sites. Thus, the proportional values of EVT cover types across the study area, and our results defined by EVT cover types (the best available cover type at the landscape scale), should be interpreted cautiously (i.e. Figure 1 pie chart). Nevertheless, field observations support the overall LANDFIRE cover type trends illustrating ponderosa pine throughout the entire study area and an increase of lodgepole pine and mixed-conifer stands in the higher elevation portions of the study area.

### Site-level fire-severity

**Figure 2:**
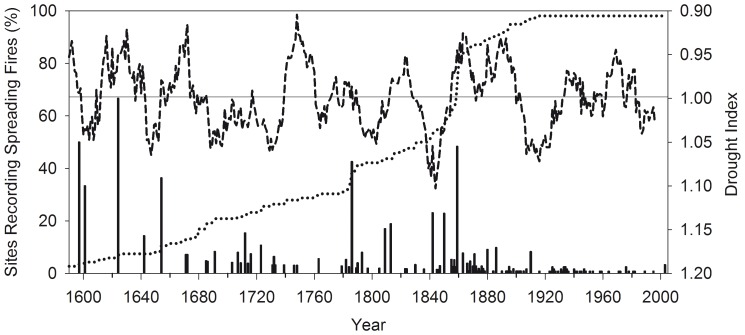
Percentage of sites recording spreading fires. Percentage of sites with spreading fires (minimum of 2 scarred trees per year at each stand-level site represented as histogram bars, n = 120 sites; left axis), sample depth of the percentage of sites available to record fires (minimum of 2 scarred trees available to record fires at each site; dotted line; left axis), and a 20-year smoothing average of a regional tree-ring drought index [60] with a mean value of 0.99 (dashed line with an inverted axis where smaller values are towards the top and indicate dry conditions; right axis).

**Figure 3:**
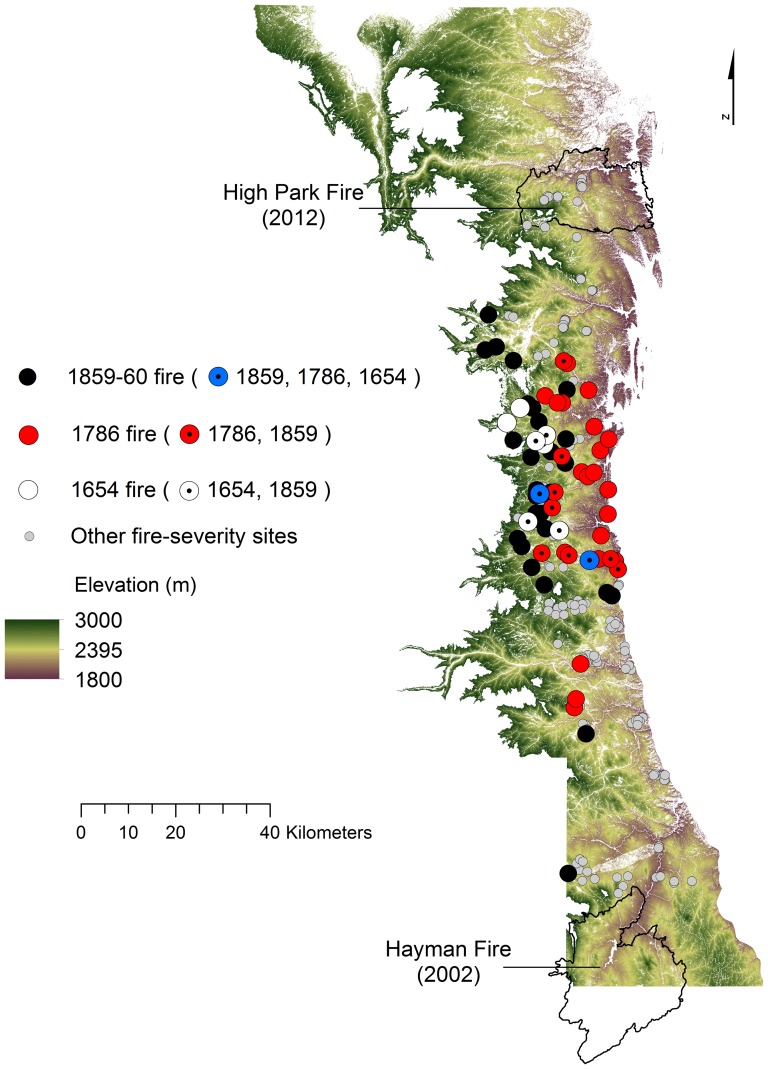
Historical fire-severity sites recording widespread fire dates in 1654, 1786, and 1859–60. Sites are shown across the elevation gradient of the study area. Symbols with centered dots indicate two or three of those fire years listed were recorded at that site. The two largest fires in recorded history for the region (Hayman Fire and High Park Fire) are shown for spatial comparison.

A total of 7680 tree cores and 1262 fire-scar samples were used to delineate fire dates and fire severity across all sites. Across 120 stand-level sites, there was evidence of 322 spreading fires (fires with ≥2 trees scarred per site) between 1597 and 1995 with only 71 (22%) of the spreading fire dates unique to one site; all other fire dates were recorded at two or more sites. Fire years of 1654, 1786, and 1859–60 were particularly extensive with 36%, 43% and 48% of the available recorder sites (with ≥2 fire-scarred trees) recording each fire year, respectively (Figure 2). These fires were recorded at multiple sites that extended over 9 km (1654), 7.5 km (1786) and up to 30 km (1859–60) distance away from one another (Figure 3). Other widespread fire dates recorded at four or more sites (recording on average 22% of the available recorder sites per year; ≥2 trees scarred per site) were 1624, 1712, 1793, 1809, 1813, 1842, 1850–52, 1857, 1863, 1866, 1871, 1880–81, 1886, and 1910 (Figure 2; multiple years indicate difficulty differentiating the exact fire year due to dormant season fires and/or missing rings before or after scarring).

**Figure 4:**
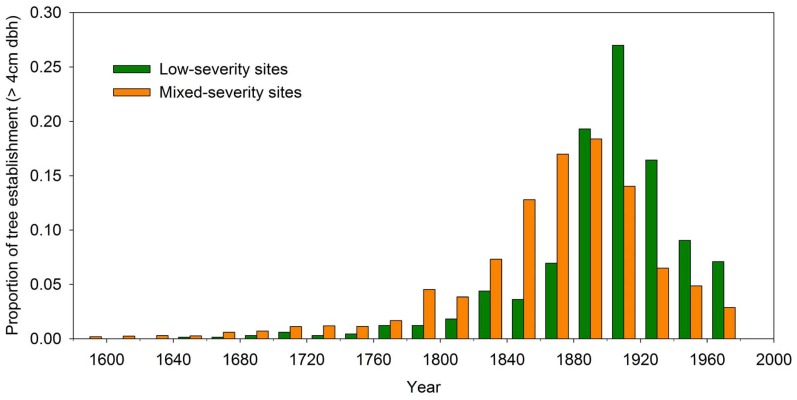
Proportion of tree establishment dates. Tree establishment (≥4 cm diameter) in 20-year bins for low-severity (green) and mixed-severity (orange) sites sampled at the stand-scale (120 sites). Total number of trees included are 663 and 5703, respectively.

Historical fire severity regime was delineated at individual sites using 297 fires that occurred prior to 1920 (pre-fire exclusion) and tree age data (remnant and establishment ages). Eighteen (7.8%) sites were characterized by only low-severity fires and 214 (92.2%) were characterized by mixed-severity fires. Across the montane region, the majority of trees established between 1820 and 1920 (66% of tree ages; Figure 4) primarily because of past mixed-severity fires during this period (this study; and also see [44]–[45], [48], [50]–[51], [53], [55]). Low-severity sites tended to have younger trees than mixed-severity sites (average date of tree establishment of 1907 compared to 1873, respectively; Figure 4). The low-severity fire regime was most common in pure ponderosa pine sites (12 sites; ≥80% of the canopy trees as ponderosa pine), but also occurred in mixed-conifer sites (6 sites with co-dominance of ponderosa pine and Douglas-fir). Sites with the historical low-severity regime tended to occur at lower elevations than sites with mixed-severity fires with median elevations of 2101 m and 2445 m, respectively.

**Figure 5:**
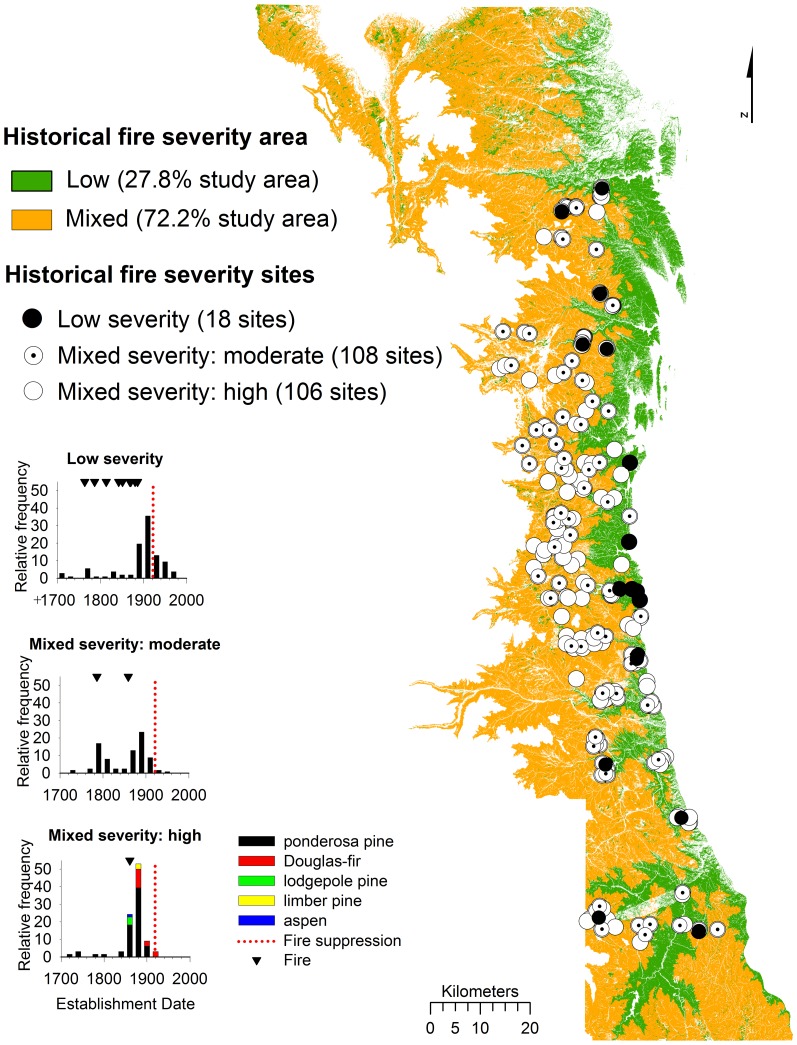
Distribution of 232 sites with historical (pre-1920) evidence of low-severity and mixed-severity fires. Sites with historical evidence of mixed-severity fires are differentiated with evidence of low- and moderate-severity fire (no evidence of high-severity fire; 108 sites) and low- to high-severity fire (106 sites). Areas mapped as historical low-severity fire only (27.8%) and mixed-severity fire (72.2%) are shown in green and orange, respectively. Three example graphs are also shown to illustrate the evidence (remnant trees, tree establishment and spreading fires) used to classify sites with evidence of low and mixed-severity fire effects at individual sites.

Of the 214 sites with evidence of mixed-severity fire (Figure 5), 108 sites had evidence of low and moderate-severity fires (no evidence of high-severity fire) and 106 sites had evidence of high-severity fires (evidence of up to ∼50 ha patches of high-severity in some stand-level sites). Interpretation of the spatial extent of historical fire severity is limited by our sampling unit size (sites ranged between ∼3–232 ha in size). Nevertheless, there is strong evidence that in most years before 1915 fires were much more extensive than the size of a single site (only 15.5% of the fire years were unique to one site; 46 of 297 fire dates; Figure 2). Many sites burned at moderate or high severity during the same year with an average of 1.1 km distance between sites (and up to 7 km distance between nearest sites burning in the same year with evidence of higher-severity fire). Evidence of mixed-severity fires occurred in all dominant cover types sampled, including sites of pure (≥80% of the canopy trees) lodgepole pine (20 sites) and ponderosa pine (89 sites), as well as mixed-conifer types with dominance of aspen (6 sites), lodgepole pine (16 sites), Douglas-fir (40 sites), and ponderosa pine (43 sites). Across sites classified with mixed-severity fire regimes, moderate- and high-severity fire effects were evident in all dominant cover types including pure lodgepole pine (8 and 12 sites) and ponderosa pine (48 and 41 sites), as well as mixed-conifer types with dominance of aspen (2 and 4 sites), lodgepole pine (8 and 8 sites), Douglas-fir (21 and 19 sites), and ponderosa pine (18 and 25 sites), respectively.

### Landscape-level historical fire severity

Elevation and slope steepness were the most important predictive variables delineating locations of only low-severity versus mixed-severity sites. The CART model with the best predicted occurrence of sites with historical low- and mixed-severity fire had a PCC of 80.6% (187 of 232 sites predicted correctly) and an AUC value of 0.77 for the cross-validation test. The model indicated low-severity sites tended to occur at or below 2263 m, or on slopes equal or less than 4 degrees above 2263 m (17 of 18 low-severity sites fit this model, 94.4%). Mixed-severity sites occurred across a broad topographic gradient of the montane zone, but tended to occur on slopes greater than 4 degrees above 2263 m (170 of 214 mixed-severity sites fit this model, 79.4%). Mixed-severity sites with evidence of high-severity fire tended to occur at higher elevation, on greater slope steepness, and at greater distances from streams compared to sites with evidence of only low-severity fires. There were no strong generalizable differences between locations of mixed-severity sites with evidence of low- to high-severity fire and those without evidence of high-severity fire (only low- and moderate-severity fires). Cover type was not a significant predictor of fire severity.

**Figure 6:**
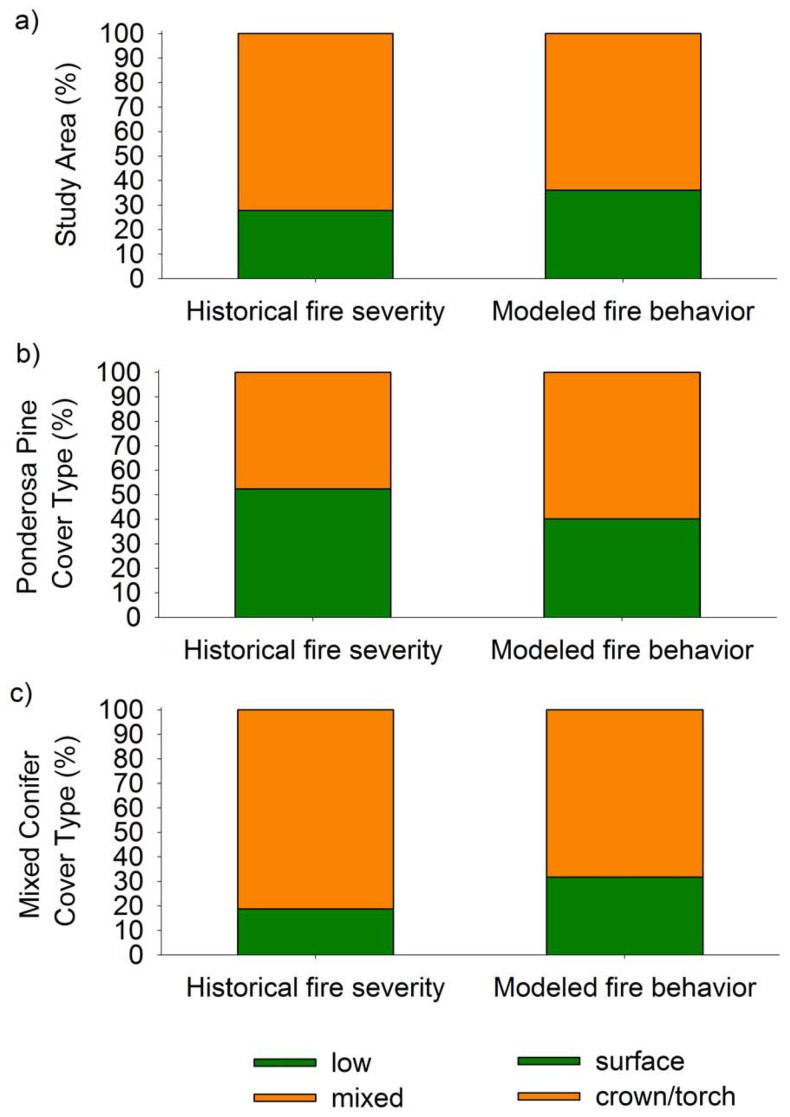
Percent study area and cover type classified with historical fire type and modeled fire behavior. A comparison of the percentage of the a) study area, b) ponderosa pine cover type, and c) mixed conifer cover type classified as historical fire (low-severity only or mixed-severity) and modeled current fire behavior (surface or crown/torch) under extreme weather conditions (99^th^ percentile for 1964–2007).

The best-fit model from the CART analysis (described above) was used to map historical fire severity across the montane study area (564,413 ha). Across all cover types, 27.8% (156,198 ha) of the area was mapped with an historical regime of low-severity fire and 72.2% (406,173 ha) was mapped as mixed-severity fire (Figure 6a). Within the low-severity fire regime (27.8% of the study area) almost equal areas were classified by LANDFIRE EVT as ponderosa pine (45.4%) and mixed conifer (48.8%), intermixed with grassland/shrubland (5.7%). In comparison, areas mapped as the historical mixed-severity fire regime (72.2% of the study area) were classified with a lower percentage of ponderosa pine (16%) and grassland/shrubland (2.7%), and a higher percentage of mixed conifer (81.3%) cover types. Of areas mapped as pure ponderosa pine, 52.4% was classified as low-severity and 47.6% as mixed severity fire (Figure 6b). Of areas mapped as mixed conifer types, 18.8% was classified as low-severity and 81.2% as mixed severity fire (Figure 6c). The decrease in pure ponderosa pine dominance above 2263 m is consistent with the elevation shift in co-dominance with other species, primarily Douglas-fir, in the upper montane zone.

### Comparison of historical fire severity to observed fire severity

**Figure 7:**
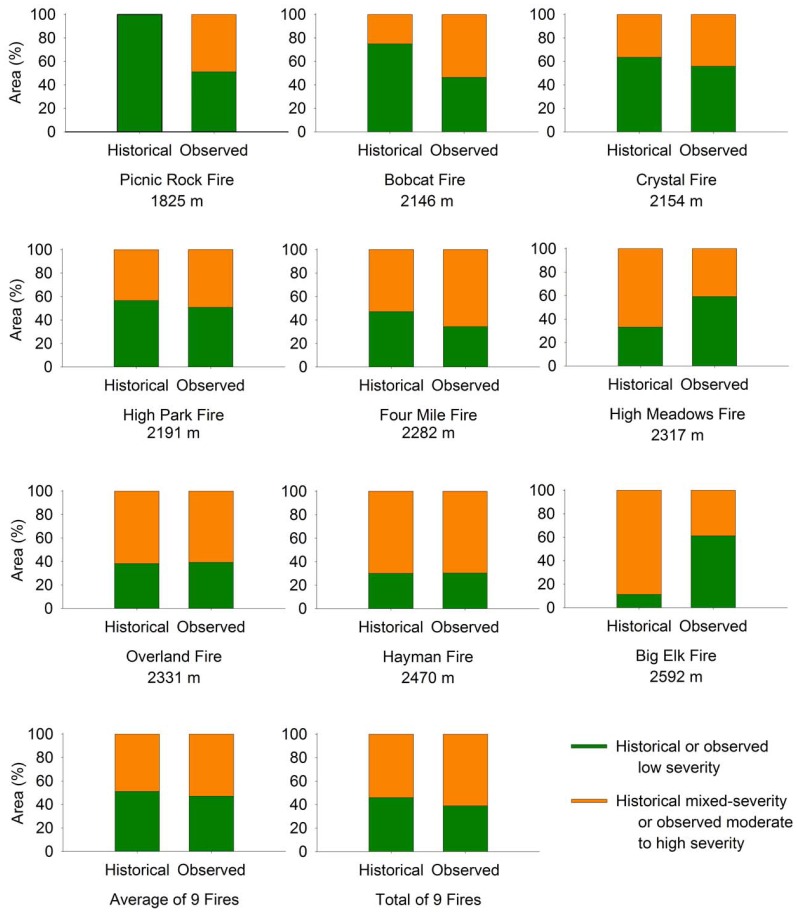
Comparisons of the historical and observed fire severities for nine recent fires (2000–2012). The comparison shows the proportion of low- (unburned/low and low) and mixed- (moderate and high) severity fire within the perimeter of nine recent fires (2000–2012), and the average and total (all pixels) proportions of fire severities across all nine fires in the study area. The median elevation for each fire is given under the fire name.

For the nine recent fires in the study area since 2000 (Table 2; Figure 1), there was a 4% difference between the average observed (47%) and historical (51%) areas of low-severity fire severity, and a 7% difference between the total observed (39%) and historical (46%) areas of mixed-severity fire (Figure 7). The greatest differences between observed (MTBS) and expected (historical) low-severity fire was at the lowest elevations (median elevations <2155 m; Figure 7). Three fires (Picnic Rock Fire, Bobcat Fire, Crystal Fire) had lower than expected observed low-severity fire compared to the historical fire severity map (*X^2^* value of 6.39 for Bobcat Fire and 5.49 for Crystal Fire, *p*<0.05; the *X^2^* test was invalid for the Picnic Rock Fire with no pixels classified as historical mixed-severity fire). For the remaining six fires there were either no significant differences in low-severity fire (High Park Fire, Four Mile Fire, Overland Fire, Hayman Fire, *X^2^* value of 1.04, 2.84, 1.56, and 2.43, respectively, *p*>0.05) or higher than expected low-severity fire observed compared to the expected historical fire severity map (High Meadows Fire and Big Elk Fire, *X^2^* values of 5.14, and 8.25, respectively, *p*<0.05).

### Verification of modeled fire behavior

Overall, we found a positive association between modeled wildfire behavior (fireline intensity and percentage crown fire) and MTBS severity class (Table S1; Figures S1 and S2). The exception was the Picnic Rock Fire, for which the modeled percentage crown fire was consistently very low across observed severity classes. This is not surprising, as the Picnic Rock Fire occurred at the lower elevation grassland ecotone with over 50% of the fire classified as unburned/low or low severity and only 21% as high severity that occurred primarily in grassland fuel types (73% of high-severity area). Overall, severely burned areas in observed fires of variable fire severities (Table 2) corresponded to higher modeled fireline intensity and proportion of crown fire (Table S1), providing confidence in the model of potential wildfire behavior and its comparison with historical fire severity.

### Comparison of historical fire severity to modeled current fire behavior

Across the entire montane study area present-day potential fire behavior was 36.1% surface and 73.9% crown (1.3% torch and 62.6% crown) fire occurrence under extreme (99^th^ percentile) weather conditions (Figure 6a). Historical fire severity for the entire study area was mapped as 27.8% low-severity only and 72.2% mixed-severity fire regimes. For the pure ponderosa pine cover type, under the extreme fire weather scenario (99^th^ percentile), present-day fuels predicted 40.2% of the area would burn as surface fire and 59.8% as crown (3.1% torch and 56.7% crown) fire (Figure 6b). For the mixed conifer cover type, the extreme fire weather scenario and present-day fuels predicted 31.8% as surface and 68.2% as crown (0.7% torch and 67.5% crown) fire (Figure 6c).

**Figure 8:**
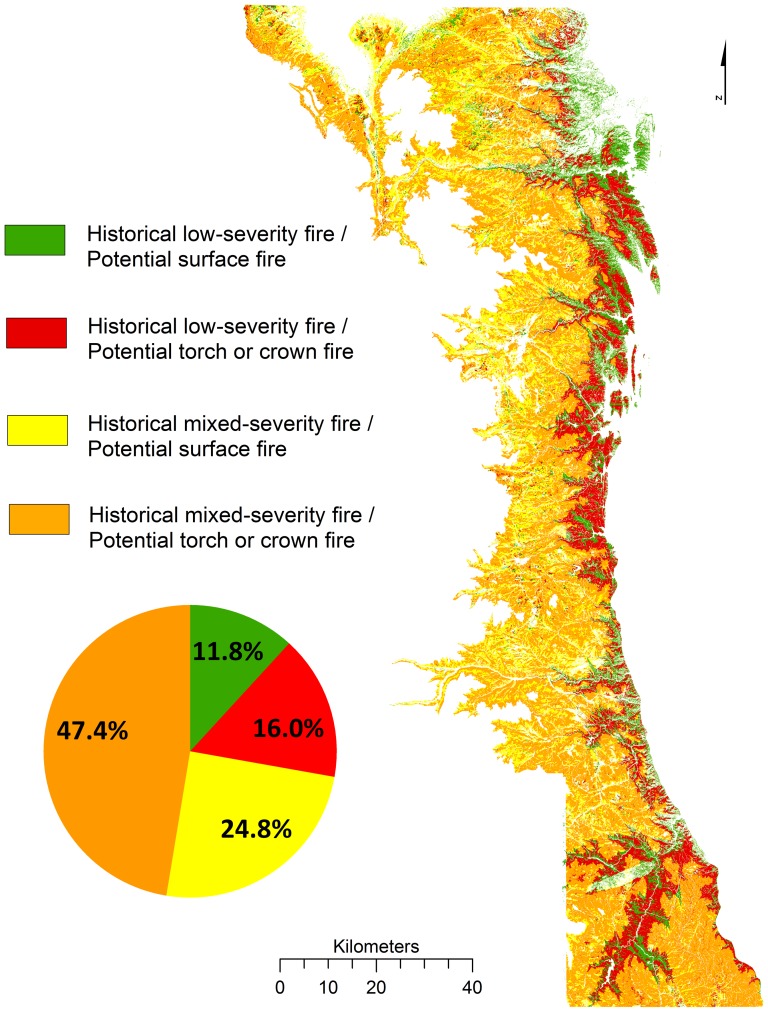
Historical fire severity overlaid with a model of fire potential under extreme (99^th^ percentile weather). The areas and proportion of the study area mapped as historical low-severity fire with current potential for surface fire (green), historical low-severity fire with current potential for torch or crown fire (red), historical mixed-severity with current potential for surface fire (yellow), and historical mixed-severity with current potential for torch or crown fire (orange). Current potential fire behavior is modeled under extreme (99^th^ percentile) weather (1964–2007).

Almost 12% (66,394 ha) of the entire study area showed little change in the low-severity fire regime (Figure 8 – green), based on a high spatial coincidence of historical low-severity and present-day potential for surface fire under extreme (99^th^ percentile) weather conditions. These areas were predominantly in the lower montane zone (mean 2136 m elevation and 12 degree slope steepness), and had an average modeled fireline intensity of 14,020 kW/m. About 16% of the entire montane study area (Figure 8 – red) is now susceptible to crown fire but historically had lower-severity fire. These areas were also concentrated in the lower montane zone (mean 2177 m elevation and 16 degree slope steepness), had an average modeled fireline intensity of 43,620 kW/m, and were classified by LANDFIRE EVT as grassland or shrubland (0.5%), lodgepole pine (5.2%), ponderosa pine (43.7%), and mixed conifer (50.7%). Of areas mapped only as the historical low-severity fire regime (27.8% and 156,198 ha of the study area; Figure 8 – green and red combined), 42.5% of the area showed little change in the fire regime (Figure 8 – green), whereas 57.5% had the potential for higher-severity fire (Figure 8 – red).

Above the lower montane zone (>2263 m), the historical and modeled fire behavior under extreme conditions showed high variability from surface to crown fire, as expected in a mixed-severity fire regime. Areas above 2263 m that were mapped as historical mixed severity fire, but today have potential for surface fire and crown fire were 24.8% and 47.4% of the entire montane study area, respectively (Figure 8 – yellow and orange). Of areas mapped only with the historical mixed-severity fire regime (72.2% and 406,173 ha of the study area; Figure 8 – yellow and orange), 34.4% has a low probability of crown fire today (mapped as potential for surface fire in Figure 8 (yellow); average fireline intensity of 15,861 kW/m), whereas 65.6% of the area has a high probability of crown fire activity today (mapped as potential for torch or crown fire in Figure 8 (orange); average fireline intensity of 42,290 kW/m).

## Discussion

A key finding of our study of fire regime changes in the montane forest zone of the Colorado Front Range is that only 16% of the total study area recorded a shift from historical low-severity to a higher potential for crown fire today. This area of increased fire severity occurs in over half (57.5%) of the area mapped with the historical low-severity fire regime and is concentrated in the lower montane zone. A substantial portion (42.5%) of the area mapped as the historical low-severity fire regime (11.8% of the study area) shows little change in the fire regime (Figure 8), and is expected to support only surface fire even under extreme (99^th^ percentile) weather conditions based on our modeling of present day fuels. Both areas occur primarily in the lower montane zone below 2263 m, but the areas with little change in the low-severity fire regime are on average slightly lower in elevation and slope steepness and closer to grasslands and ravine drainages than areas with higher potential for crown fire today. Observations from recent large wildfires and modeling of potential fire behavior under extreme weather conditions are consistent with the historical evidence of a varied fire regime of primarily low-severity fires at the lowest elevations to a mixed-severity fire regime at higher elevations in montane forests. Three regional fires predominantly in the lower montane forests showed the greatest differences between the observed (MTBS of recent large fires) and expected (historical fire severity) areas burned by low-severity fire. However, the differences between the observed and expected proportions of low-severity fire for the six other fires were non-significant, or showed higher than expected low-severity fire.

A decline in fire frequency over the past 100 years leading to substantial increases in stand density is supported only for the lowest elevations of forest below approximately 2200 m in the Colorado Front Range [13]–[14], [45], [51], [60], [86]. These areas were characterized mainly by frequent (average return intervals <30 years) low-severity fires that maintained open forests by killing mostly juvenile trees, resulting in low densities of mature trees. Greater proximity to grassland in lower elevation areas probably promoted more frequent fire due to more abundant fine, herbaceous fuels [62]. The cessation of formerly frequent fires coincides with increased stand densities broadly throughout the lower montane zone. This pattern is especially evident below 2200 m, but also occurred at some sites at higher elevations on less steep slopes most likely where montane grasslands occurred. However, overall this represents a relatively small proportion of the montane forest of the northern Colorado Front Range (27.8% of the study area is mapped with the historical low-severity fire regime).

The dominant (72.2% of the 564,413 ha study area) historical fire regime of the northern Colorado Front Range consisted of a mixed-severity regime in which stand structures were shaped primarily by moderate-severity (46.5% of sites) and high-severity (45.7% of sites) fires; only 7.8% of the sites recorded predominantly low-severity fires. At higher elevation (>2263 m), spreading fires were typically less frequent (>30 year fire intervals), and had varied fire effects (mixed-severity) that included non-stand replacing fire to canopy-replacement fire both within sites and across broad landscapes, often in relation to topographic variability. Many sites experienced intervals between successive widespread fires that would have been sufficient for conifer seedlings to reach sizes that would survive low-severity surface fires. Specifically, evidence of high-severity fire tended to occur further from grasslands and on steeper sites than those with evidence of only low-severity fire, although there is overlap between sites that show moderate- or high-severity effects particularly above the lower montane zone. Evidence of mixed-severity fires occurred in all dominant cover types sampled including sites of pure (≥80% of the canopy trees) lodgepole pine and ponderosa pine, as well as mixed-conifer types with dominance of aspen, lodgepole pine, Douglas-fir, and ponderosa pine. Consistent site- to landscape-scale evidence indicates an historical mixed-severity fire regime in which moderate- and high-severity fire effects shaped current forest age structures in the mid- and upper montane zone [13], [46], [51], [53] (and this study).

Most spreading fires recorded at our sites extended beyond a single site (88% of 322 fires with ≥2 trees scarred per site from 1597–1995). Moderate- and high-severity fires prior to the 20^th^ century were documented at many sites, as illustrated by fires in 1654, 1786 and 1859–60 that extended at least 7–30 km between nearest sampling sites, are within the HRV for montane forests at a regional scale (Figure 3). For example, the spatial extent of sites recording fires in the same year is within the spatial scale of the largest modern wildfires on record in the study area (e.g. High Park Fire and Hayman Fire). Our ability to interpret the spatial extent of historical fire severity patches from individual fires is limited by our sampling unit size (3 ha–232 ha), subsequent fire events, and sample depth (although >20% of our sites record fires since 1700), but the evidence indicates mixed-severity fires occurred in patches up to at least 200 ha, with evidence of approximately 50 ha of high-severity in some sites. Direct comparison of patch sizes of fires is difficult given the presence of major highways and other land uses that have fragmented fuel continuity at a landscape scale. It is highly likely that variance in patch size of historical fires was great, similar to modern fire observations, rendering quantitative comparisons of rather limited value. Nevertheless, high-severity fire and high-stand densities are within the HRV for the mid- and upper montane forests (e.g. [44], [51], [53]–[54], this study), which needs to be taken into account when forest management goals consider restoring forests to pre-fire-exclusion conditions.

In the Colorado Front Range, at higher elevations (>2263 m) mixed-severity fire regimes appear to have been the predominant fire regime historically and today. The modeled potential wildfire behavior under current fuel conditions and extreme fire weather shows a mixed-severity fire regime throughout the montane forests of the Colorado Front Range. High fireline intensity and active crown fire are within the HRV of a mixed-severity fire regime particularly under extreme conditions, but would vary in extent of intensity and canopy mortality depending on existing conditions. Site-level evidence indicates high overlap in biophysical conditions that support moderate and high-severity fires (this study and [52]). These results provide a snapshot of the expectations into the near future for fire behavior in the Colorado Front Range. The advantage of comparing historical and observed fire severity with fire behavior modeling across the study area under realistic, but also extreme, weather conditions is that it provides an important present-day comparison to the past. Recent large wildfire years in the Colorado Front Range are not without precedent and similar events occurred historically (i.e. 1654, 1786, 1859–60), under similar exceptional (interannual to multi-decadal scale) climate conditions [61]. The largest fires since the year 2000 – the Hayman and High Park Fires – were both characterized by up to 98–99th percentile conditions for both temperature and relative humidity. The situation will likely be exacerbated under climate change; under the 2007 IPCC A2 scenario for the study area, the 99^th^ percentile temperatures of the past are expected to become the 39^th^ percentile temperatures by 2064–2099 (also see [87]). Thus, the range of weather conditions during local to more widespread fire years in the last 300+ years likely represents a similar range of potential fire-climate conditions presently, and informs expectations of potential fire behavior that may occur under severe, yet increasingly more common weather conditions.

We recognize that each of our spatial datasets (mapping of historical, observed, and potential fire) have their own set of limitations. For example, distance to grassland was not a predictor in the best-fit model used to map the historical fire severity landscape. However, our prior research indicates areas adjacent to grasslands experience more frequent fires than sites farther from grasslands, owing to the proximity to prevalent fine fuels that increase the sensitivity of fire activity to interannual climate variability [62]. Thus, the historical model likely under-estimates the amount of low-severity fire in some areas at higher elevation within or adjacent to grasslands where we have limited or no sample sites (e.g. high elevation grassland areas in the northern portion of the study area - Figures 1 and 8). This could explain why some of the study area (Figure 8) shows a shift from historical mixed-severity fire to potential low-severity fire in the contemporary forest even under the extreme weather scenario. The observed fire behavior dataset (MTBR Thematic Burn Severity) has its own limitations. To classify fire severity into ‘high’ ‘moderate’, and ‘low’ severity classes, image analysts determine thresholds in dNBR values based on visual interpretation of imagery, field plot data, and expert knowledge. Though dNBR is sensitive to initial vegetation conditions [88], alternatives such as RdNBR are less parsimonious and in practice may not capture burn severity with greater accuracy than dNBR [89]. Also, while the fire behavior modeling from FlamMap operates on a fixed set of fuels and weather conditions, the historical severity model is based on the fire regime over a long period of time during which fires of different severity have the opportunity to burn. Because of these limitations, we report proportional area of landscape-level changes rather than finer-scale interpretations, and emphasize broad trends evident from multiple lines of inquiry. Additionally, while our model was based on the most recently available fuels data from LANDFIRE, characterizing vegetation changes across the landscape due to wildfire and management through 2010, mortality from recent insect outbreaks, for example, are not reflected. Inferring the effect of fuel and fire behavior changes in our model due to tree mortality from insects is challenging as such fuel changes are: 1) highly dependent on the timing and severity of the outbreak, 2) not characterized well in standard fuel models, and 3) not validated for fire behavior as few empirical studies have examined how the timing and extent of insect outbreaks in montane forests affects observed fire behavior.

### Implications for fuels management and ecological restoration

A clear delineation of the spatial extent of past fire regime types is a major concern for ecosystem managers in the context of wildfire risk and ecological restoration [90]–[92]. Evidence from previous studies in ponderosa pine and other montane forests in the Colorado Front Range have shown that the historical fire regime was variable over time and space, represented by a mixed-severity regime [44], [46]–[48], [50]–[54]. Goals of ecological restoration and wildland fire hazard mitigation are both compatible with management practices, like prescribed fire and thinning to reduce fuels, below approximately 2200 m in our study area, which experienced the greatest increase in fire severity, and likely fuels, since fire exclusion [93]. Disturbance from grazing and logging as well as periods of favorable climate probably also contributed to increased tree establishment in the late 19^th^ and early 20^th^ century, but seedling survival clearly depended on the long fire-free periods. Even at low elevations, however, some sites had historical fire regimes dominated by infrequent rather than frequent fires (e.g. steep and north-facing slopes [86]). These infrequent fires, inferred to be high-severity fires from age structure data, killed high percentages of trees within a fire perimeter, and promoted the establishment of naturally dense forest patches [44], [51], [86]. This suggests that some of the areas (e.g. north-facing and steep slopes) with potential for crown fire today may support characteristic fire behavior and are not necessarily out of the HRV even in the lower montane zone.

Mixed-severity fire regimes where spreading fires occurred at lower frequencies (>30 year fire intervals) are less clearly candidates for thinning than are low-severity fire regimes and a cautious approach to restoration efforts has been recommended [15], [40]. In areas naturally characterized by lower frequencies of moderate- to high-severity fires, fuel reduction through prescribed fire and thinning will likely not achieve both ecological restoration and fire hazard mitigation goals. Restoration thinning treatments will not return the fire regime to one of low severity across the Front Range montane zone, which was historically predisposed to periodic fires of varying severities, and are of questionable effectiveness in preventing severe wildfires [2]. Where extreme fire behavior appears within HRV, high-severity fire may largely be explained by extreme weather conditions (for example, high winds and low humidity during severe drought) rather than quantity of woody fuels [38]. This is illustrated by the 2002 Hayman Fire in Colorado in which more than 24,000 ha burned at high severity throughout ponderosa pine and mixed-conifer forests of variable stand structures in a single day [94]. The fire-weather conditions presented here (99^th^ percentile for the late 20^th^ century and early 21^st^ century) represent only the projected average expected conditions by the mid-21^st^ century, which is a pressing issue for existing and future WUI development [95] and fire management [2]. Learning from recent experiences where wildfire damage has been high in the WUI [2], along with considering the costs of suppressing future wildland fires (i.e. reducing forest resiliency, unsustainable federal costs to society [1]) and the sociopolitical expectations of wildland fire management are critical for managing future fire risks and forest integrity.

For the ponderosa pine cover type, an increasing number of studies in the Pacific Northwest [15], [96]–[100] have also documented fire regimes of variable severity. These studies and ours illustrate the importance of collecting evidence on the severity of past fires (i.e. remnant tree and cohort ages, growth releases, tree mortality) because fire severity cannot be reliably interpreted from fire interval data alone, which has often the approach applied to the study of fire history in ponderosa pine forests. Although current implementation of national programs (Collaborative Forest Landscape Restoration Program) and databases (LANDFIRE) recognize variation in fire regimes within the same cover type across different geographical regions, fire regimes of a cover type within a region can still be considered relatively uniform. This is problematic in the Front Range and potentially elsewhere where the historical fire regime varied from low-severity at the lowest elevations to mixed-severity over most of the montane zone regardless of forest type. Additionally, the accuracy of the current cover type (EVT) layer for the region is low. Thus, the spatial and proportional reliability of the LANDFIRE classifications of fire regimes and vegetation do not accurately reflect the historical or current landscape, and therefore will not be highly effective in prioritizing locations for fire management and forest restoration objectives. Further corroboration of these issues are also documented at a national scale (see p. 19 in [92]) “The accuracy of various aspects of the LANDFIRE data is questionable, even when used at intended scale…Without accurate data, many assumptions and actions based on this data will be compromised. There is a need for more realistic and accurate depiction of where wildland fire hazard/risk actually occurs across the country, which can be used to base decisions upon.”

Our study, along with prior studies, shows that an assumption of fire regime uniformity is not valid for the ponderosa pine and other montane cover types in the Colorado Front Range and elsewhere (i.e. [15]). These findings challenge ecologists and managers to re-consider the degree of variation in fire regimes within broadly distributed forest types. Thus, management efforts to create large areas of open woodlands in the higher elevation areas of the montane zone of the Colorado Front Range would not be consistent with historical fire regimes and stand structures.

## Supporting Information

Figure S1 **MTBS fire severity classification (observed) and median fire line intensity (modeled).** The comparison was used as a measure of verification of the modeled fire behavior.
(PDF)

Figure S2 **MTBS fire severity class (observed) and percentage of crown fire (modeled).** The comparison was used as a measure of verification of the modeled fire behavior.
(PDF)

Table S1 **Summary table of biophysical factors for each observed fire and potential fire model in analysis.**
(XLSX)

Table S2 **List of plot-level sites.** The fire severity classification for each site was documented from forest structure and fire scars in the field using an existing protocol [52].
(PDF)
