# Supplementary material for: Historical, Observed, and Modeled Wildfire Severity in Montane Forests of the Colorado Front Range
Source: PLoS One. 2014 Sep 24;9(9):e106971. doi: 10.1371/journal.pone.0106971 (PMC4175072; doi:10.1371/journal.pone.0106971)
Supplement: Figure S1 — MTBS fire severity classification (observed) and median fire line intensity (modeled). The comparison was used as a measure of verification of the modeled fire behavior. (PDF) [file pone.0106971.s001.pdf]

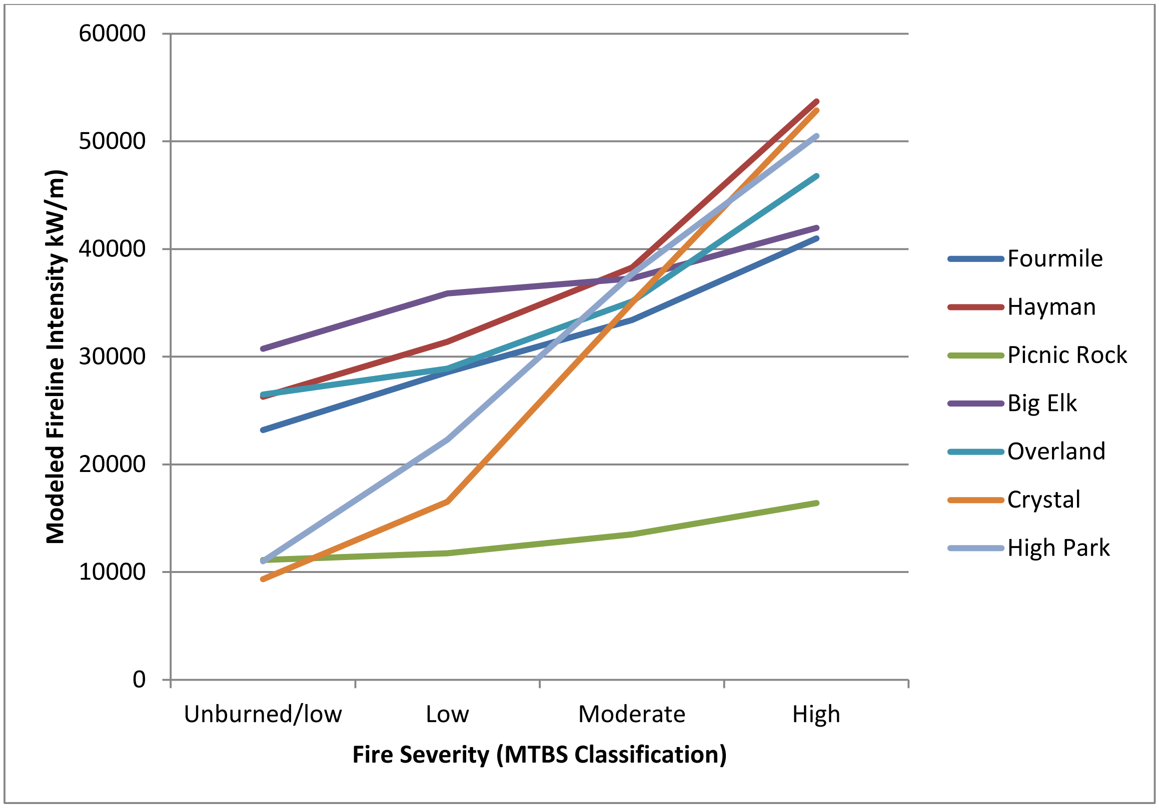

Figure S1. MTBS fire severity classification (observed) and median fire line intensity (modeled). The comparison was used as a measure of verification of the modeled fire behavior.
