# Supplementary material for: Historical, Observed, and Modeled Wildfire Severity in Montane Forests of the Colorado Front Range
Source: PLoS One. 2014 Sep 24;9(9):e106971. doi: 10.1371/journal.pone.0106971 (PMC4175072; doi:10.1371/journal.pone.0106971)
Supplement: Table S2 — List of plot-level sites. The fire severity classification for each site was documented from forest structure and fire scars in the field using an existing protocol [52]. (PDF) [file pone.0106971.s004.pdf]

Table S2. List of plot-level sites. The fire severity classification for each site was documented from forest structure and fire scars in the field using an existing protocol [52].

Plot Site No.	Fire severity classification	Latitude	Longitude
**1**	mixed	40.6717	-105.3672
**2**	mixed	40.6718	-105.3669
**3**	low	40.6702	-105.3662
**4**	mixed	39.9343	-105.3409
**5**	mixed	39.9354	-105.3393
**6**	mixed	40.6277	-105.3780
**7**	mixed	40.6635	-105.3688
**8**	mixed	40.6635	-105.3685
**9**	mixed	40.6616	-105.3688
**10**	mixed	40.6571	-105.3693
**11**	mixed	40.6557	-105.3700
**12**	mixed	40.6559	-105.3703
**13**	mixed	40.6569	-105.3693
**14**	mixed	40.6548	-105.3663
**15**	mixed	40.6365	-105.4497
**16**	mixed	40.6376	-105.4525
**17**	mixed	40.6378	-105.4520
**18**	mixed	40.6363	-105.4495
**19**	mixed	40.6362	-105.4304
**20**	mixed	40.6370	-105.4278
**21**	mixed	40.6368	-105.4274
**22**	mixed	40.6348	-105.4270
**23**	mixed	40.6301	-105.4607
**24**	low	40.6284	-105.4614
**25**	low	40.6285	-105.4615
**26**	mixed	40.6303	-105.4615
**27**	mixed	40.4814	-105.3695
**28**	mixed	40.4795	-105.3683
**29**	mixed	40.4792	-105.3685
**30**	low	40.4800	-105.3696
**31**	mixed	40.4586	-105.3382
**32**	mixed	40.4575	-105.3401
**33**	mixed	40.4577	-105.3405
**34**	mixed	40.4590	-105.3392
**35**	mixed	40.4005	-105.4083
**36**	mixed	40.4011	-105.4059
**37**	mixed	40.4015	-105.4057
**38**	mixed	40.4019	-105.4078
**39**	mixed	40.4017	-105.4073
**40**	mixed	40.3921	-105.4119
**41**	mixed	40.3924	-105.4098
**42**	mixed	40.3926	-105.4096
**43**	mixed	40.3928	-105.4118

**44**	low	40.3879	-105.4101
**45**	mixed	40.3866	-105.4117
**46**	mixed	40.3868	-105.4121
**47**	mixed	40.3890	-105.4120
**48**	low	40.3799	-105.3532
**49**	mixed	40.3814	-105.3526
**50**	mixed	40.3804	-105.3543
**51**	mixed	39.8515	-105.4227
**52**	mixed	39.8607	-105.4440
**53**	mixed	39.7833	-105.4218
**54**	mixed	39.8467	-105.4456
**55**	mixed	39.8386	-105.4270
**56**	mixed	39.8588	-105.3888
**57**	mixed	39.8555	-105.4099
**58**	mixed	39.8387	-105.4067
**59**	mixed	39.8580	-105.3734
**60**	mixed	39.8592	-105.3610
**61**	mixed	39.8494	-105.3615
**62**	mixed	39.8470	-105.3798
**63**	mixed	39.8967	-105.2707
**64**	mixed	39.8945	-105.2725
**65**	mixed	39.7788	-105.2563
**66**	mixed	39.7703	-105.2557
**67**	mixed	39.6323	-105.2380
**68**	mixed	39.6431	-105.2117
**69**	mixed	39.6363	-105.2184
**70**	mixed	39.6381	-105.2225
**71**	mixed	39.6290	-105.2243
**72**	mixed	39.7485	-105.3243
**73**	mixed	39.7571	-105.3273
**74**	mixed	39.7609	-105.3320
**75**	mixed	39.7622	-105.3475
**76**	mixed	39.7577	-105.3598
**77**	mixed	39.7428	-105.3636
**78**	mixed	39.7475	-105.3502
**79**	mixed	39.7468	-105.3538
**80**	mixed	39.6310	-105.3593
**81**	mixed	39.6207	-105.3487
**82**	mixed	39.6101	-105.3478
**83**	mixed	39.6097	-105.3605
**84**	mixed	39.6640	-105.3659
**85**	mixed	39.6638	-105.3743
**86**	mixed	39.6752	-105.3782
**87**	mixed	39.6759	-105.3727
**88**	mixed	39.5326	-105.1612
**89**	mixed	39.5291	-105.1595
**90**	mixed	39.5243	-105.1553
**91**	mixed	39.5237	-105.1573

**92**	mixed	39.5357	-105.1819
**93**	mixed	39.5329	-105.1842
**94**	mixed	39.7352	-105.2484
**95**	mixed	39.7341	-105.2402
**96**	mixed	39.8128	-105.2843
**97**	mixed	39.8111	-105.2931
**98**	low	39.8199	-105.2801
**99**	mixed	39.8166	-105.2722
**100**	mixed	39.8281	-105.2922
**101**	low	39.8266	-105.2784
**102**	mixed	39.3489	-105.3878
**103**	mixed	39.3486	-105.3468
**104**	mixed	39.3680	-105.3577
**105**	mixed	39.3379	-105.2735
**106**	mixed	39.3399	-105.2470
**107**	mixed	39.3348	-105.3283
**108**	mixed	39.3940	-105.1721
**109**	mixed	39.3397	-105.1670
**110**	mixed	39.3307	-105.1328
**111**	mixed	39.3309	-105.0896
**112**	mixed	39.8814	-105.3006
